# Neuraminidase 1 (NEU1) promotes proliferation and migration as a diagnostic and prognostic biomarker of hepatocellular carcinoma

**DOI:** 10.18632/oncotarget.11778

**Published:** 2016-09-01

**Authors:** Guojun Hou, Gang Liu, Yuan Yang, Yixue Li, Shengxian Yuan, Linghao Zhao, Mengchao Wu, Lei Liu, Weiping Zhou

**Affiliations:** ^1^ The Third Department of Hepatic Surgery, Eastern Hepatobiliary Surgery Hospital, Second Military Medical University, Shanghai, China; ^2^ Key Laboratory of Systems Biology, Institute of Biochemistry and Cell Biology, Shanghai Institutes for Biological Sciences, Chinese Academy of Sciences, Shanghai, China; ^3^ Institute of Biomedical Sciences, Fudan University, Shanghai, China; ^4^ The Department of Hepatic Surgery, Eastern Hepatobiliary Surgery Hospital, Shanghai, China

**Keywords:** neuraminidase 1, hepatocellular carcinoma, prognosis, migration, proliferation

## Abstract

Hepatocellular carcinoma (HCC) is among the most malignant cancers worldwide, lacking biomarkers for subtyping and the reliable prognostication. Herein, we report a novel biomarker, NEU1 (neuraminidase 1), is up-regulated in most samples of HCC. The diagnostic value of NEU1 was evaluated by ROC, and the AUC (area under curve) reached 0.87 and 0.96 in two independent datasets, respectively. The survival differences of HCC patients with high or low expression of NEU1 were statistically significant, and a significant correlation between NEU1 expression and clinical information including stage, differentiation, AFP and embolus were observed. NEU1 expression, at both the mRNA and protein levels, were also higher in the portal vein tumor thrombus than tumor tissues. We also measured the proliferation and migration ability of two HCC cell lines following NEU1 interference and over-expression. Migration and proliferation rate were increased in NEU1 high expression groups. Moreover, gene expression studies identified pathways significantly associated with NEU1 expression. Among them, all the genes involved in spliceosomepathway were up regulated in NEU1-high group. In summary, our work identified NEU1 as a novel biomarker for both diagnosis and prognosis in HCC, and one of the most altered pathway of NEU1 is spliceosome.

## INTRODUCTION

Hepatocellular carcinoma (HCC) is the fifth common cancer worldwide, with 470,000 new cases and 420,000 death in China in 2015 [1]. The incidence of HCC in China is largely related to the prevalence of hepatitis B/C virus [2]. Not only is HCC is an aggressive tumor which often metastasizes, but HCC is also a highly heterogeneous disease [3], with a 3-year survival rate of it less than 10% [4]. Thus, new diagnostic and prognostic tools are urgently needed.

In the last decade, efforts have been devoted to identify novel biomarkers for HCC using large cohorts of tumors. This approach led to an abundance of prognostic genes/proteins. For example, serum AFP [5] is a widely used biomarker for the early identification of HCC based its high accuracy, low cost and ease of testing. This modality of testing is now an important biomarker in the clinical diagnosis of this disease [6]. Similarly, enzymes are usually highly expressed in metabolism-related cells, such as liver cells, and metabolic enzymes were reported to be significantly altered in HCC and other cancers. Glucose represents a critical component of the proliferation of most cancer cells, and overexpression of PKM2 (pyruvate kinase M2)[7] is responsible for the increased phosphate pentose pathway; Similarly, mutation of IDH1/2 are responsible for the accumulation of 2-HG (2-hydroxyglutarate)[8], which was recognized as an oncometabolite [9], while changes to serine metabolism in breast cancer and SCD-CoA in HCC have also been previously identified [10]. However, the number of reported enzymes whose expression are altered in HCC remain relatively limited, particularly those with diagnostic, prognostic and therapeutic potential.

In the present study, we demonstrate thatNEU1is an oncogene, upregulated in HCC and may present a novel diagnostic marker. Our analysis demonstrated that NEU1correlates with important clinicopathological features of HCC, while functional studies demonstrated that siRNA-mediated silencing of NEU1 is associated with decreased proliferation and migration of HCC cell lines. Pathway analysis revealed that spliceosomal function in addition to other cancer-related pathways were altered following changes in NEU1 expression.

## RESULTS

### NEU1 is a potential diagnostic marker for HCC

**Figure 1:**
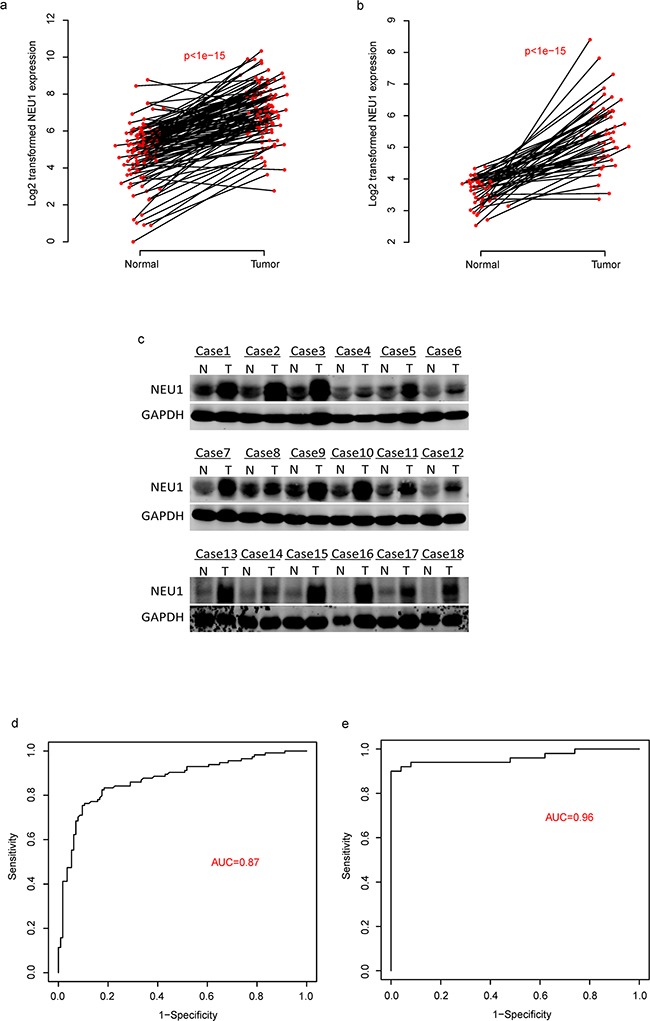
The diagnostic effect of NEU1 in HCC The expression of NEU1 is up-regulated in tumor tissues compared to the normals in two datasets **a-b.** on mRNA level and protein level **c.** And the diagnostic effect of NEU1 were evaluated by ROC curve in two independent datasets **d-e.**

Using RT-qPCR, we evaluated the expression levels of NEU1 in 114 solid tumor tissues and normal liver tissues (clinical information of the patients is available in Supplementary Table S1). We found that NEU1 is significantly overexpressed in HCC tumors as compared to normal liver (Figure 1a). We validated these findings using a validation cohort of 50 pair of normal-tumor samples (Figure 1b) according to RNA-seq analysis (GSE77314). We also assessed the protein expression of NEU1 in tumor-normal paired samples from 18 patients. In conjunction with our mRNA expression data, NEU1 was also significantly higher at the protein level in most tumors (Figure 1c). These results demonstrate that NEU1 is overexpressed in HCC.

We subsequently evaluated the diagnostic utility of NEU1 in these two datasets. According to the receiving operating character (ROC) curve (Figure 1d), the area under curve (AUC) reached 0.87 and 0.96 in the discovery and validation cohort, respectively, indicating that is a potential valuable biomarker for the diagnosis of HCC (Figure 1e).

### NEU1 is a prognostic marker in HCC

**Figure 2:**
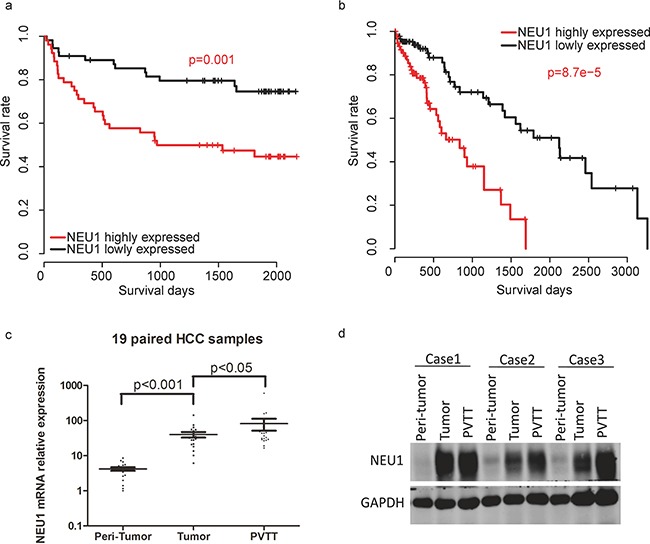
The prognostic effect of NEU1 in HCC Patients with high NEU1 expression has a relatively poor overall survival rate in both our **a.** and TCGA **b.** datasets. The mRNA **c.** and protein **d.** level of NEU1 in portal vein tumor thrombus (PVTT) is higher than tumor tissues.

In order to evaluate the prognostic significance of NEU1 in HCC, we analyzed the survival of patients with high and low expression of NEU1 based on 114 RT-qPCR results. The survival rate of in the NEU1-low subgroup was significantly better than the rate observed in the NEU1-high group (NEU1-low and NEU1-high group was divided according to the median NEU1 expression level of samples, Figure 2a). To validate these findings, we analyzed the correlation between NEU1 expression levels and survival in 269 HCC samples from the TCGA dataset. This analysis was consistent with our dataset thus confirming the prognostic utility of NEU1 (Figure 2b). We also evaluated the expression of NEU1 in 19 normal-tumor-portal vein tumor thrombus (PVTT) paired samples, and found that the highest expression of NEU1 was observed in PVTT tissues (Figure 2c), indicating the possible pro-metastatic role of NEU1. The protein levels of NEU1 measured by Western blot in PVTT tissues, was also consistent with this observation (Figure 2d).

### The correlations of NEU1 expression and clinical information

We analyzed both the clinical characteristics and the NEU1 gene expression to characterize the role of NEU1 in HCC. The relationship between clinicopathological features of the disease associated with NEU1-high versus –low tumors are shown in Table 1 (Fisher's exact test). We found that 87.0% of patients presented T1-T2 stage in the NEU1-high group while only 69.8% were T1-T2 in the NEU1-low group (p=0.001). Moreover, 78% of patients existed embolus in portal vein in the NEU1-high group versus only 51% in the NEU1-low group (p=0.005). Similar trends were observed in regards to the rate of differentiation (low differentiation 31.5% versus 4%; p=0.0002) in the high versus low NEU1 group. The other observations including microvascular embolus (0.008) and AFP (0.008), daughter nodule (p=0.04), and metastasis status (p=0.003) were also significantly different between these two groups. These results suggest that NEU1 correlates with important clinical observations and may be a proliferation and metastasis-related gene in HCC.

**Table 1:** The association between NEU1 expression and clinical information

Variables	NEU1-low	NEU1-high	p-value
**Extrahepatic metastases**			**0.0031**
No	39	26	
Yes	5	17	
**Differentiate**			**0.00022**
Low(1-2)	17	2	
High(3-4)	37	51	
**Primary Tumor satge**			**0.0011**
Low(1-2)	47	31	
High(3-4)	7	22	
**Diameter**			0.053
Small(<5cm)	30	19	
Large(>5cm)	24	35	
**Daughter nodule**			**0.036**
No	47	37	
Yes	7	16	
**PVTT**			**0.0048**
No	42	27	
Yes	12	26	
**HBsAg**			0.46
Neg	12	8	
Pos	42	45	
**BCLC**			0.46
Neg	11	8	
Pos	41	45	
**Microvascular embolus**			**0.0075**
No	43	29	
Yes	11	24	
**AFP**			**0.0081**
Low(<20)	25	12	
High(>20)	28	43	

The NEU1-low and NEU1-high group is identified according to the median expression level of NEU1

* Some clinical information is missing

### NEU1 effected the proliferation and migration of HCC cell lines

**Figure 3:**
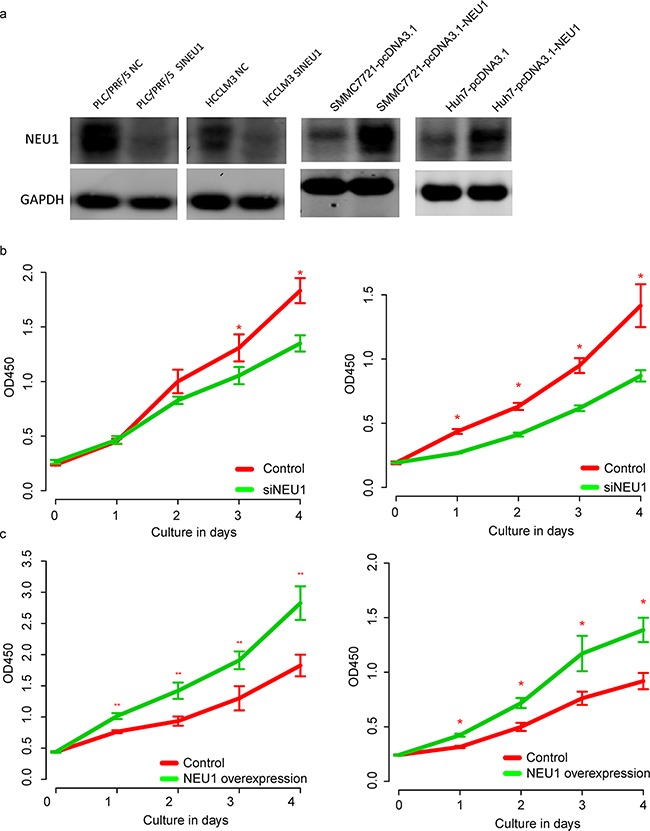
The proliferation promoting effect of NEU1 After knock down of NEU1 in HCCLM3 and PLC/PRF/5 (**a**, left panel) cells lines, the proliferation rate of both cell line were decreased after NEU1 depression (**b**, left, HCCLM3; right, PLC/PRF/5); After over-expression of NEU1 in SMMC7721 and Huh7 cell lines (**a**, right panel), the proliferation rate was significantly enhanced (**c**, left, SMMC7721; right, Huh7).

We also performed functional studies to evaluate the role of NEU1 *in vitro*. Using two different HCC cell lines, MHCCLM3 and PLC/PRF/5, we silenced NEU1 using siRNAs and assessed the impact on proliferation (Figure 3a). Knockdown of NEU1 significantly reduced proliferation as compared to control cell lines (Figure 3b). We also over-expressed NEU1 in another two cell lines (Figure 3a) and measured the proliferation rate. The OD450 value of NEU1 expression groups was significantly higher (Figure 3c), demonstrating that NEU1modulates the proliferation rate of HCC cells.

**Figure 4:**
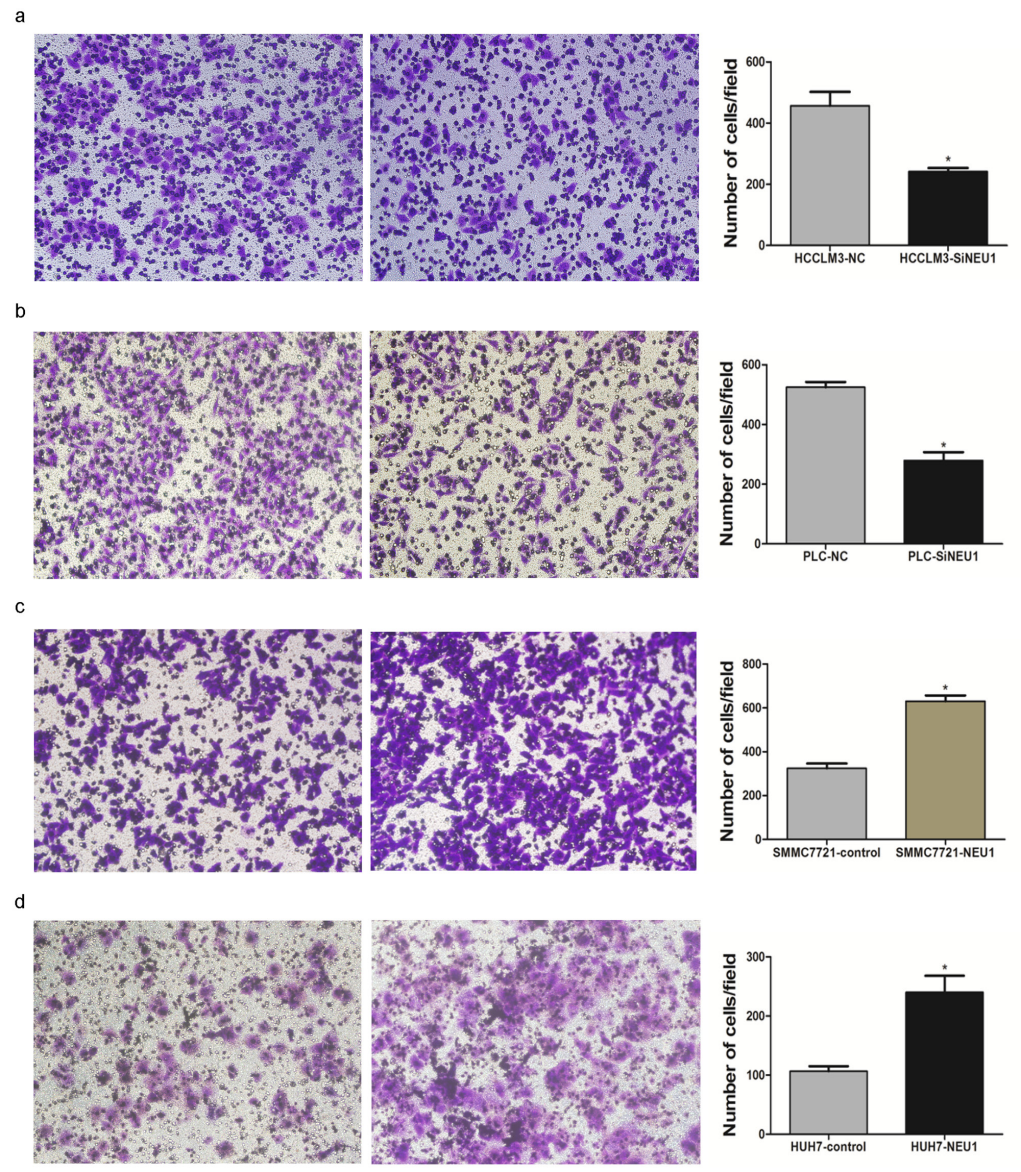
The migration promoting effect of NEU1 After knock down of NEU1, the migration rate were significantly decreased in both MHCCLM3 **a.** and PLC/PRF/5 **b.** cell lines with Transwell study. Over expression of NEU1 significantly enhanced the migration rate of SMMC7721 **c.** and Huh7 **d.** cell lines.

Our previous results demonstrated that the overexpression of NEU1 was associated with PPVT and embolus, and therefore we also evaluated the impact of NEU1 expression on cell using a transwell experiment. Following siRNA-mediated suppression of NEU1 for 48 hours, the number of migrated cells of control group was 2-folds higher than that observed in the NEU1 knock down group (Figure 4a–4b), in both MHCC-LM3 and PLC/PRF/5 cell lines. The migration rate of the NEU1 over expressed SMMC7721 and Huh7 cell lines was about 2-folds than the controls (Figure 4c–4d), indicating that NEU1 may promote metastasis of HCC cells.

### Potential mechanisms of NEU1-induced proliferation and metastasis

**Figure 5:**
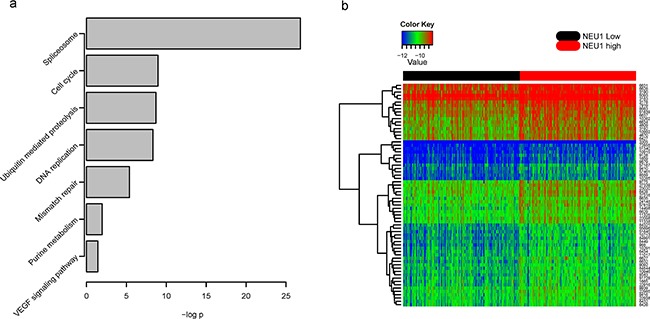
Pathway analysis of differential genes between NEU1-high and NEU1-low group Several cancer related signaling pathways were enriched **a**, and most genes involved in splicesome were highly expressed in NEU1-high group **b.** The –log p indicates the -log 10 transformed p value of the corresponding pathway. The row names of the heat map is Entrez gene IDs of the corresponding genes.

To further investigate the potential signaling pathway which contributes to NEU1-mediated proliferation and migration of HCC cells, we identified differentially expressed genes between NEU1-highversus NEU1-low expressing tumors across a cohort of 269 patients from the TCGA dataset (according to the median expression level of the samples used). A total of 2321 genes were significantly differentially expressed between these groups. We performed pathway analysis using DAVID, and found that spliceosome, cell cycle, ubiquitin mediated proteolysis, DNA replication, mismatch repair, purine metabolism, VEGF signaling pathway were significantly enriched (Figure 5a). Of these pathways, we noticed that spliceosome was most significantly enriched and most genes enriched in this pathway was up-regulated in NEU1 highly expressed group (Figure 5b), suggesting that the alteration of NEU1 is associated with the expression of spliceosome genes.

## DISCUSSION

The highly heterogeneous nature of hepatocellular carcinoma makes this diagnosis particularly challenging. In the present work we evaluated the expression of NEU1 in two independent cohorts, demonstrating that NEU1 was significantly upregulated in tumor tissue. We found that this gene is a valuable diagnostic marker in distinguishing normal and tumor tissues, highly expression in tumor tissues on both the mRNA and protein levels, with AUC reaching 0.85 and 0.96 in two independent datasets. It is also demonstrated that the expression level of NEU1 in tumor tissue is a prognostic marker for HCC, associated with the survival time and specific clinicopathological features of HCC. Moreover, we functionally validated the effect of NEU1 expression, demonstrating that loss of NEU1 leads to reduced proliferation and migration, respectively.

NEU1 is a lysosomal neuraminidase enzyme that cleaves a terminal sialic acid residue from substrates such as glycoproteins and glycolipids. While NEU1 has been shown to be upregulated in cancer cells in previous reports, there existed controversy regarding the effect of NEU1 in different tumors. NEU1 was reported to suppress metastasis both *in vitro* and *in vivo* in human colon cancer [11], however in ovarian cancer, knock down of NEU1 inhibited the proliferation, apoptosis, and invasion of tumor cells [12]. Our results suggests that NEU1 promotes metastasis and proliferation of hepatocellular carcinoma cell lines *in vitro*, however further research is needed to characterize the tumor-specific role of this gene.

To investigate the potential mechanism underlyingNEU1-mediated migration and proliferation, we performed KEGG pathway enrichment analysis evaluating differentially expressed genes between NEU1-high and –low groups. Our results indicated that the most significantly affected pathway was the spliceosome pathway. The spliceosome is a ribonucleoprotein complex that controls (alternative) splicing, and is involved in cell cycle, invasion, metastasis and angiogenesis [13], thus supporting our *in vitro* findings. Recent studies have shown that targeting the spliceosome reduced the proliferation rate of cancer cell lines [14], and triggers mTOR blockade and autophagy [15]. These results suggested that NEU1 may effect proliferation and metastasis of HCC through changes in the spliceosomal function, and identifies a novel therapeutic avenue in this disease.

In conclusion, our study reported the identification of a novel prognostic and diagnostic marker, NEU1, which promotes HCC cell proliferation and migration through spliceosome pathway, which may provide a new target for the treatment of HCC in future.

## MATERIALS AND METHODS

### TCGA data processing

Clinical information and gene expression data (data level 3) were downloaded from TCGA. The scaled estimate were downloaded and normalized using the quartile method. Two groups were divided according to the median expression level of each gene, and survival differences were evaluated using the R package “survival” [16].

### RNA extraction and quantitative real-time PCR (RT-QPCR)

All patients whose tissue were used in this study provided written informed consent. Total RNA was isolated using Trizol (Invitrogen, CA) according to the manufacturer's protocol. RNA quality and quantity was evaluated by Nanodrop 2000 (Thermo Scientific, USA), and first-strand cDNA was synthesized from 2μg of total RNA using random primers and M-MLV Reverse Transcriptase (Invitrogen, CA). Expression of candidate genes was evaluated using real-time polymerase chain reaction (RT-PCR)performed using an ABI PRISM 7900 sequence detector (Applied Biosystems, Carlsbad, CA) and SYBR Green PCR kit (Applied TaKaRa, JA), with 18s as the endogenous control. Each sample was tested in duplicate, and the relative mRNA expressions were calculated based on the Ct values and were normalized via 18s expression.

### Cell culture and transfection

Liver cancer cell lines PLC/PRF/5, SMMC-7721, HCC-LM3 and Huh7 were purchased from Cell Bank of Type Culture Collection of Chinese Academy of Sciences. Cell lines were maintained at 37°C in an atmosphere containing 5% CO2 in Dulbecco's modified Eagle's medium supplemented with 10% fetal bovine serum. For in vitro study, PLC/PRF/5, HCC-LM3 were transfected with siRNA (Biotend, Shanghai, People's Republic of China) against NEU1 to knock down NEU1, and SMMC-7721, HuH7 were transfected with pcDNA-NEU1(GenePharma, Shanghai, People's Republic of China)to overexpress NEU1 using Lipofectamine2000 transfection reagent (Invitrogen, USA) according to the manufacturer's instructions. siRNA-control and pcDNA were used as a negative control.

### Western blot

Total protein was extracted using RIPA Lysis Buffer and PMSF (Thermo Scientific, USA) according to the manufacturer's instructions, and centrifuged at 12,000rpm for 15 minutes. Protein concentrations were measured using the bicinchoninic acid assay. Antibody dilutions were 1:500 for the NEU1 antibody (Abcam, Cambridge, MA) and 1:10000 for GAPDH (Santa Cruz Biotechnology) and immunocomplexes were incubated with the fluorescein-conjugated secondary antibody. Antibody binding was detected with an Odyssey infrared scanner (Li-CorBiosciences, Inc.)

### Cell migration and proliferation analysis

Migration assays were performed using the transwell filter chambers (Costar, Corning, NY) according to the manufacturers' instructions. Briefly, 1×10^5^cells were resuspended in serum-free medium, and added into the top chamber. Medium with 10% FBS was added to the lower chamber. Following 12 hours of incubation, cells on the lower surface of the membrane were stained, photographed, and counted using a microscope in six random fields per field for each group. These experiments were performed in triplicate. For proliferation assay, Cells were seeded into 96-well plates (3000/well) and cell viability was determined by Cell Counting Kit-8 (Dojindo Laboratories, JA) every 24 hours.

### Data analysis

Survival differences among groups were estimated using the R package “survival”, and the receiving operating character (ROC) curve were calculated and drawn with R package “ROCR” [17].

Gene expression differences between NEU1 highly and lowly expressed tumors were evaluated using student t-test, and false discovery rate (FDR) were calculated with bonferroni, and genes with FDR less than 0.01 were retained for further analysis. KEGG pathway enrichment analysis based on genes identified were analyzed with DAVID tools [18] (The Database for Annotation, Visualization and Integrated Discovery v6.7).

## SUPPLEMENTARY MATERIALS TABLE




