# Supplementary material for: Neuraminidase 1 (NEU1) promotes proliferation and migration as a diagnostic and prognostic biomarker of hepatocellular carcinoma
Source: Oncotarget. 2016 Sep 1;7(40):64957–66. doi: 10.18632/oncotarget.11778 (PMC5323129; doi:10.18632/oncotarget.11778)
Supplement: Supplementary file 1 [file oncotarget-07-64957-s001.pdf]

Neuraminidase 1 (NEU1) promotes proliferation and migration as a diagnostic and prognostic biomarker of hepatocellular carcinoma

SUPPLEMENTARY TABLE

Supplementary Table S1: Clinical information of the 114 patients.

See Supplementary File 1
