# Supplementary material for: Neuraminidase 1 (NEU1) promotes proliferation and migration as a diagnostic and prognostic biomarker of hepatocellular carcinoma
Source: Oncotarget. 2016 Sep 1;7(40):64957–66. doi: 10.18632/oncotarget.11778 (PMC5323129; doi:10.18632/oncotarget.11778)
Supplement: Supplementary file 2 [file oncotarget-07-64957-s002.docx]

Table S1 Clinical information of the 114 patients.

| **Variables** | **NEU1-low** | **NEU1-high** | **p-value** | **sample number** |
| --- | --- | --- | --- | --- |
| **Gender** |  |  | 1 |  |
| Male | 52 | 50 |  | 102 |
| Female | 5 | 5 |  | 10 |
| **Reccurence** |  |  | 0.521281186 |  |
| No | 27 | 22 |  | 49 |
| Yes | 18 | 20 |  | 38 |
| **metastasis** |  |  | **0.00306845** |  |
| No | 39 | 26 |  | 65 |
| Yes | 5 | 17 |  | 22 |
| **Dead** |  |  | **0.001226381** |  |
| No | 0 | 13 |  | 13 |
| Yes | 44 | 2 |  | 46 |
| **Age** |  |  | 0.805676533 |  |
| Young(<60) | 0 | 0 |  | 0 |
| Old(>60) | 2 | 1 |  | 3 |
| **HBV_history** |  |  | 0.458076217 |  |
| No | 8 | 11 |  | 19 |
| Yes | 46 | 42 |  | 88 |
| **differentiate** |  |  | **0.00022101** |  |
| Low(1-2) | 17 | 2 |  | 19 |
| High(3-4) | 37 | 51 |  | 88 |
| **Primary Tumor satge** |  |  | **0.00106358** |  |
| Low(1-2) | 47 | 31 |  | 78 |
| High(3-4) | 7 | 22 |  | 29 |
| **diameter** |  |  | 0.052747777 |  |
| Small(<5cm) | 30 | 19 |  | 49 |
| Large(>5cm) | 24 | 35 |  | 59 |
| **Membrane** |  |  | 0.191994128 |  |
| No | 27 | 12 |  | 39 |
| Yes | 15 | 18 |  | 33 |
| **daughter nodule** |  |  | **0.035861698** |  |
| No | 47 | 37 |  | 84 |
| Yes | 7 | 16 |  | 23 |
| **PVTT** |  |  | **0.004724939** |  |
| No | 42 | 27 |  | 69 |
| Yes | 12 | 26 |  | 38 |
| **Hep1** |  |  | 0.143500088 |  |
| Neg | 7 | 13 |  | 20 |
| Pos | 47 | 40 |  | 87 |
| **HBsAg** |  |  | 0.458076217 |  |
| Neg | 12 | 8 |  | 20 |
| Pos | 42 | 45 |  | 87 |
| **CK18** |  |  | 0.420648816 |  |
| Neg | 22 | 17 |  | 39 |
| Pos | 31 | 36 |  | 67 |
| **BCLC** |  |  | 0.457102226 |  |
| Neg | 11 | 8 |  | 19 |
| Pos | 41 | 45 |  | 86 |
| **microvascular embolus** | |  | **0.007521366** |  |
| No | 43 | 29 |  | 72 |
| Yes | 11 | 24 |  | 35 |
| **AFP** |  |  | **0.008085699** |  |
| Low(<20) | 25 | 12 |  | 37 |
| High(>20) | 28 | 43 |  | 71 |
